# Intraperitoneal laparoscopic single-site lymph node dissection in modified supine position during laparoscopic radical nephroureterectomy

**DOI:** 10.1093/jscr/rjae368

**Published:** 2024-06-05

**Authors:** Ming Xiong, Menghao Zhou, Yi Luo, Huiling Jiang, Gallina Kazobinka, Yajun Xiao, Teng Hou

**Affiliations:** Department of Urology, South China Hospital, Medical School, Shenzhen University, No. 1 Fuxin Road, Longgang District, Shenzhen 518116, China; Department of Urology, South China Hospital, Medical School, Shenzhen University, No. 1 Fuxin Road, Longgang District, Shenzhen 518116, China; Department of Urology, Union Hospital, Tongji Medical College, Huazhong University of Science and Technology, 1277 Jiefang Avenue, Jianghan District, Wuhan 430022, China; Department of Surgery, Distinct HealthCare, Shenzhen 518061, China; Department of Urology, South China Hospital, Medical School, Shenzhen University, No. 1 Fuxin Road, Longgang District, Shenzhen 518116, China; Urology Unit, La Nouvelle Polyclinique Centrale de Bujumbura, Bujumbura 378, Burundi; Department of Urology, Union Hospital, Tongji Medical College, Huazhong University of Science and Technology, 1277 Jiefang Avenue, Jianghan District, Wuhan 430022, China; Department of Urology, South China Hospital, Medical School, Shenzhen University, No. 1 Fuxin Road, Longgang District, Shenzhen 518116, China; Department of Urology, Union Hospital, Tongji Medical College, Huazhong University of Science and Technology, 1277 Jiefang Avenue, Jianghan District, Wuhan 430022, China

**Keywords:** upper tract urothelial carcinoma, ureteral neoplasms, nephroureterectomy, lymph nodes excision, supine position

## Abstract

Technique modifications that aim to improve ergonomics of the surgical procedure without repositioning the upper tract urothelial carcinoma patients remain a challenge to urologists. We offer a novel technique to perform intraperitoneal laparoscopic single-site radical nephroureterectomy and pelvic lymph nodes dissection/retroperitoneal lymph nodes dissection in a supine position. Our novel technique is feasible and offers a significant improvement in operative efficiency, particularly in patients with locally advanced disease.

## Introduction

Radical nephroureterectomy with bladder cuff excision is a standard procedure for treating upper tract urothelial carcinoma (UTUC). For patients with high-grade or muscle-invasive diseases, lymph node dissection (LND) is becoming a necessary treatment strategy [1, 2]. However, it is difficult to perform laparoscopic radical nephroureterectomy (LRNU) and pelvic lymph node dissection (PLND) concurrently. In addition, laparoscopic retroperitoneal lymph node dissection (RPLND) is challenging using traditional approaches. Here, we describe our technique of intraperitoneal LRNU with bladder cuff excision and PLND/RPLND in a modified supine position without patient’s repositioning for UTUC.

## Case report

Eleven patients with urothelial carcinoma in the distal ureter underwent LRNU and PLND, eight patients with tumors in the renal pelvis or upper ureter underwent LRNU and RPLND. Laparoscopic PLND or RPLND was performed according to an anatomical template-based approach described by Matin et al. [3].

**Figure 1:**
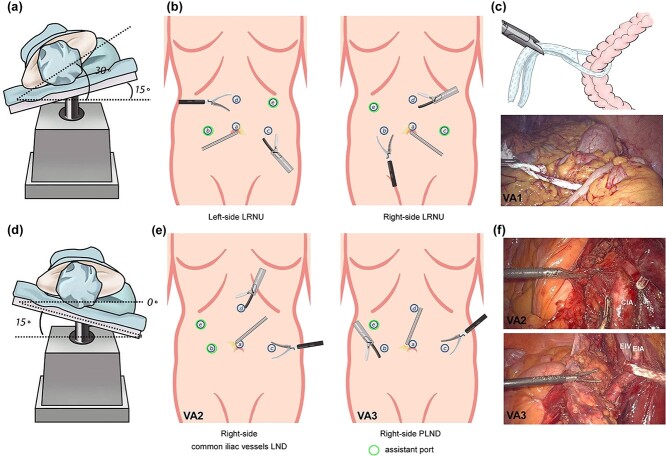
Diagram of patient positions, trocar placement, and the use of cotton cord for surgical exposure. (a) Patient position for right-side LRNU. (b) Trocar placement for LRNU. For each lesion side, the right-hand port of the surgeon is for ultrasonic scalpel. (c) A cotton cord was used to wrap around the descending colon, and a grasping forcep was inserted by port b to retract the bowel medially away from the surgical field. (d) Patient position for right-side PLND and bladder cuff excision. (e) Trocar placement for common iliac vessels LND, PLND and bladder cuff excision. (f) Visual angles for common iliac vessels LND and PLND. A cotton cord was used to sling the external iliac vessels. CIA: common iliac artery; EIA: external iliac artery; EIV: external iliac vein.

Patients were placed in a supine position with an inflatable pillow placed behind the affected flank, which provides a 20°–30° rotation of the abdomen (Fig. 1a). They were also secured to the operating table with 2-inch tapes to permit tilting. A 10-mm camera port was placed in that position (port a). Two 12-mm trocars were placed at the umbilical level and 6 cm right and left to the umbilicus (port b and c), respectively, for insertion of the operator instruments. A fourth 5-mm working port was placed below the xiphoid at the middle line which is also superior to the umbilical port (port d), and the fifth trocar position was 2 cm below the costal margin along the medioclavicular line (port e). Port e can also be placed below the costal margin along the midclavicular line to retract the liver for a right-sided nephroureterectomy (Fig. 1b).

In the first visual angle (VA1), the line of Toldt was incised. A cotton cord was used to wrap around the bowel, and a grasping forceps was inserted via port b to retract the bowel medially away from the surgical field (Fig. 1c). Then ureter was identified and dissected cephalad until reaching the renal hilum level. After ligation of the renal artery and vein with Hem-o-lock clips and consequent transaction, the kidney was released and the ureter was freed to the external iliac vessels level. Then the operating table was axially rotated so that the patient is in a Trendelenburg and complete supine position, and the laparoscope was turned caudally (VA2) for PLND, distal ureter dissection, and bladder cuff excision (Fig. 1d–e). A grasping forcepsinserted via port e was used to sling the external iliac vessels with a cotton cord (Fig. 1f). After the lateral umbilical ligament was transected, distal ureter was dissected and occluded with Hem-o-lok to avoid tumor seeding. A 1-cm margin of bladder cuff was achieved, and the cuff was excised and then sutured with 3-0 polyglyconate.

**Figure 2:**
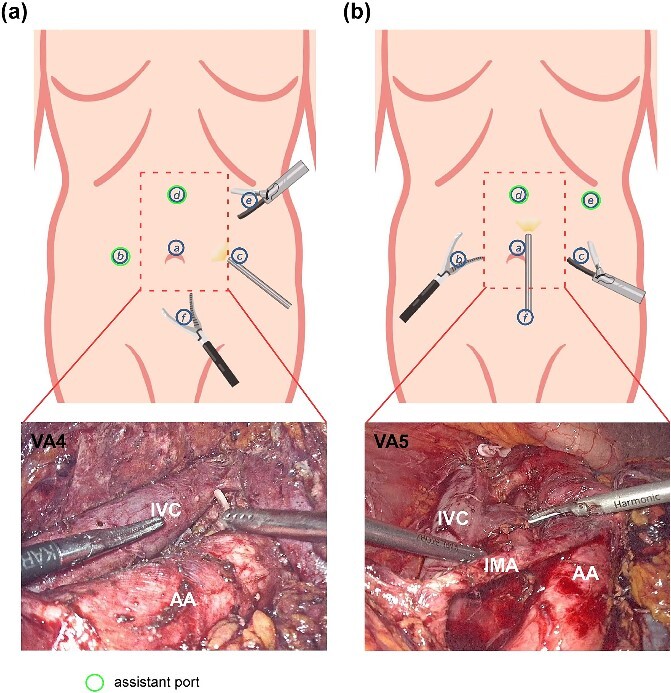
Diagram of patient positions, trocar placement, and the use of cotton cord for surgical exposure. (a) Patient position for right-side LRNU. (b) Trocar placement for LRNU. For each lesion side, the right-hand port of the surgeon is for ultrasonic scalpel. (c) A cotton cord was used to wrap around the descending colon, and a grasping forcep was inserted by port b to retract the bowel medially away from the surgical field. (d) Patient position for right-side PLND and bladder cuff excision. (e) Trocar placement for common iliac vessels LND, PLND and bladder cuff excision. (f) Visual angles for common iliac vessels LND and PLND. A cotton cord was used to sling the external iliac vessels. CIA: common iliac artery; EIA: external iliac artery; EIV: external iliac vein.

For RPLND, an additional 12-mm port was positioned 6 cm below the umbilical port (port f). The visual angles of left-side RPLND are shown in Fig. 2. VA3 is preferably used for para-aortic LND, while VA4 is preferably used for interaortocaval LND. A fan-shaped retractor was used to help expose the aorta and the inferior vena cava.

Perioperative data for patients is presented in Table 1. Four patients were found to have pelvic lymph node metastasis, while two patients have retroperitoneal lymph node metastasis. During the mean follow-up of 20 months (range, 5–42), two patients had local recurrence, and two developed distant recurrence. Intravesical recurrences occurred in four patients who underwent transurethral resection of bladder tumors.

**Table 1:** Surgical outcomes of patients

Variables	LRNU+PLND	LRNU+RPLND
	*n* = 11	*n* = 8
Mean operative time (min)	191 ± 21.6	204 ± 30.3
Blood loss (ml)	102 ± 46.9	85 ± 46.3
Postoperative hospitalization (days)	4 ± 1	5 ± 2
Mean lymph node number (*n*)	13 ± 4	15 ± 7
Intraoperative complications (*n*)	0	0
Chylous leaks (*n*)	1	2
Positive surgical margin (*n*)	0	0
Bladder recurrence (*n*)	3	1

## Discussion

The most common location of ureteral tumor is the distal segment, which is usually more invasive than renal pelvic carcinoma [4, 5]. Therefore, LND for patients with tumors in the lower third of the ureter is a vital portion of the surgical treatment. As recommended by Matin *et al*., patients with invasive distal ureteral tumors should undergo radical nephroureterectomy and PLND [3], while no publication has addressed the issue of how to perform LRNU and PLND in a single session. Our approach for the first time combines the technical advantages of LRNU in the lateral decubitus position and PLND in the supine position, which, thus, offers an efficient strategy for the performance of concurrent LRNU and PLND.

For tumor in the renal pelvis or in the upper two-thirds of the ureter, RPLND may be necessary. But there is no consensus of the most appropriate technique as it is difficult to dissect retroperitoneal lymph nodes along the aortocaval region in a traditional lateral decubitus position. Li *et al*. described a new technique of extraperitoneal LRNU and RPLND in a modified supine position [6]. Similarly, Miki *et* al. used a complete supine position that permits complete access for extraperitoneal LRNU and concomitant lymphadenectomy [7]. However, the extraperitoneal approach is suitable for all patients, including those with a history of retroperitoneal surgery, or those with preoperative imaging showing that retroperitoneal lymph nodes were enlarged and fused. In those cases, our transperitoneal approach in the supine position may provide an effective and safe alternative to the extraperitoneal approach.

Using the technique described in our case, the transperitoneal approach with a supine position permits concurrent LRNU and PLND/RPLND without any intraoperative repositioning. Moreover, the transperitoneal approach provides better exposure for the resection of enlarged and fused lymph nodes, and UTUC accompanied by severe hydronephrosis. Additionally, the transperitoneal supine position facilitates the management of bladder cuff due to the sufficient visualization compared with the lateral decubitus position. We believe that our technique is the treatment of option for patients with locally advanced disease, in whom a thorough PLND/RPLND is warranted.

## Data Availability

All data generated or analyzed during this study are included in this article. Further inquiries can be directed to the corresponding author.
